# High-throughput drug screening identifies fluoxetine as a potential therapeutic agent for neuroendocrine prostate cancer

**DOI:** 10.3389/fonc.2023.1085569

**Published:** 2023-03-13

**Authors:** Lei Chen, Yiyi Ji, Ang Li, Bo Liu, Kai Shen, Ruopeng Su, Zehua Ma, Weiwei Zhang, Qi Wang, Yinjie Zhu, Wei Xue

**Affiliations:** Department of Urology, Renji Hospital, School of Medicine, Shanghai Jiao Tong University, Shanghai, China

**Keywords:** NEPC, fluoxetine, prostate cancer, drug repurposing, high-throughput drug screening

## Abstract

**Introduction:**

Neuroendocrine prostate cancer (NEPC) is an aggressive subtype of prostate cancer with poor prognosis and resistance to hormone therapy, which has limited therapeutic approaches. Therefore, this study aimed to identify a novel treatment for NEPC and provide evidence of its inhibitory effects.

**Methods:**

We performed a high-throughput drug screening and identified fluoxetine, originally an FDA-approved antidepressant, as candidate therapeutic agent for NEPC. We carried out both in vitro and in vivo experiments to demonstrate the inhibitory effects of fluoxetine on NEPC models and its mechanism in detail.

**Results:**

Our results demonstrated that fluoxetine effectively curbed the neuroendocrine differentiation and inhibited cell viability by targeting the AKT pathway. Preclinical test in NEPC mice model (PBCre4: Ptenf/f; Trp53f/f; Rb1f/f) showed that fluoxetine effectively prolonged the overall survival and reduced the risk of tumor distant metastases.

**Discussion:**

This work repurposed fluoxetine for antitumor application, and supported its clinical development for NEPC therapy, which may provide a promising therapeutic strategy.

## Introduction

Neuroendocrine prostate cancer (NEPC) is an aggressive subtype of prostate cancer with high heterogeneity and poor differentiation. It is featured with the expression of neuroendocrine markers and the loss of the androgen receptor related pathway, and often arises under the effects of androgen deprivation therapy (1, 2). The incidence of treatment-induced NEPC has raised as a result of increasing clinical applications of androgen receptor targeting agents such as enzalutamide and abiraterone (3). The clinical features of NEPC are quite different from usual prostate adenocarcinoma, including significantly shorter median overall survival, higher incidence of lethal visceral metastases, and less sensitivity to androgen deprivation therapy or docetaxel-based chemotherapy (4).

Previous studies have reported that the neuroendocrine differentiation of prostate cancer was associated with various genetic alterations. For instance, the combined loss of *RB1* and *PTEN* facilitates lineage plasticity and metastasis, and the combined loss of *RB1* and *TP53* results in resistance to androgen deprivation therapy (5–7). Moreover, the loss of *RB1* and *TP53* increases the expression of epigenetic reprogramming factors like EZH2 and SOX2, promoting the progression of tumors and the resistance to androgen deprivation therapy. It has been reported that EZH2 inhibitors, such as GSK126 and EPZ6438, can curb cell viability and sensitize the NEPC to enzalutamide treatment (8).

In our study, we performed high-throughput screening and found fluoxetine, a Food and Drug Administration (FDA)-approved antidepressant, can effectively reduce cell viability and promote the apoptosis of NEPC. *In vitro* and *in vivo* experiments suggested that fluoxetine induced the downregulation of NE markers and prolonged overall survival of mice models by targeting the AKT pathway. We concluded that fluoxetine has the potential to be repurposed as a novel treatment for NEPC.

## Methods and materials

### Drug screening

We tested the drug libraries with a total of 7315 compounds in LASCPC-01 cell line with Cell Explorer High Throughput Screening Workstation (PerkinElmer^®^). Before the screening, cells were seeded in the CulturPlate 384-well plate (PerkinElmer^®^, 6055700) and treated with individual compounds at a concentration of 2μM. Dispensing of cell suspension or reagent was completed by Multidrop Combi (Thermo Scientific^®^, 5840330). After 72 hours of incubation, cell viability was evaluated by CellTiter-Glo Luminescent Assay (Promega^®^, G7572) according to the manufacturer’s instructions. DMSO was selected as the negative control for each compound. The cell viability corresponding to each compound will be normalized, using Cell viability (Compounds/DMSO) as the calculation method. Three replications were performed. The results of 5-HT pathway drugs in high-throughput drug screening were collected in the **Supplementary Table 1**.

### Cell line and organoid culture

The human prostate cancer cell line LASCPC-01 was purchased from the American Type Culture Collection (ATCC). LASCPC-01 cells were cultured in modified HITES medium with Hydrocortisone, human recombinant insulin, transferrin, estradiol, and sodium selenite. All the tumor cell conditional medium was supplemented with 5% fetal bovine serum (Gibco, A3161002C) and 100 U/ml penicillin-streptomycin (Thermo Fisher Scientific, 15140122). All the cells were cultured in a humidified incubator at 37°C with 5% CO_2_. The drug reagent fluoxetine was purchased from MedChemExpress (MCE, LY-110140) and was applied according to the recommended manufacturer’s protocol. Fluoxetine was pre-mixed with complete culture medium in advance and diluted to working concentrations. The plvx304-MYCN-HA-puro-CMV plasmid was purchased from the Core Facility of Basic Medical Sciences, Shanghai Jiao Tong University School of Medicine. Lentivirus was prepared using a three-plasmid packing system. PC3-NMYC^ov^ cell line was selected in 2 μg/mL puromycin for 1 week.

Murine prostate tumor tissues were collected, minced, and digested in DMEM culture medium (Gibco, 11965092) containing 0.2mg/mL collagenase (Gibco, 17100017), 0.1mg/mL DNase I (STEMCELL Technologies, 07469), 1U/mL dispase (Sangon, 78990-62-2) and 2% FBS (Gibco, 10099). Digestion was performed on a shaker for 1 hour at 37°C. Tumor cells were blocked with CD16/32 antibody for 40 minutes, and then stained with EpCam antibody (BioLegend, 2%FBS in PBS) for 1 hour on ice. Then the anti-Biotin Microbeads (Miltenyi Biotec) were added into the cell suspension for incubation at 4°C. LS columns (Miltenyi Biotec) were used for isolating EpCam (+) tumor epithelial cells with the magnetic field produced by a magnetic cell sorting (MACS) separator.

### Real time quantitative PCR

Total RNA in cells was prepared using TRIzol Reagent (Ambion, 15596026) method according to the manufacturer’s protocol. The concentration and quality were evaluated by NanoDrop 2000c spectrophotometer (Thermo Fisher Scientific). Complementary DNA (cDNA) was synthesized with HiScript III RT SuperMix for qPCR (Vazyme, R122-01) from reverse transcription. Then, RT-qPCR was performed using ChamQ Universal SYBR qPCR Master Mix reagent (Vazyme, Q121-02). Relative gene mRNA expression level was detected using the 2^-ΔΔCq^ method and was normalized to the reference gene and indicated control group.

### Western blot analysis

The whole protein was extracted from cells using RIPA lysis buffer with 1% PMSF and 1% Protease inhibitor cocktail added (Beyotime Biotechnology, ST506, P1112). The concentration of protein was evaluated using BCA protein assay kit (Thermo Fisher Scientific, 23225). At least 10μg protein samples were loaded onto 10% SDS/PAGE gel. Prestained Protein Ladder (Thermo Fisher Scientific, 26616) was used as protein markers. After running, the proteins were transferred to 0.45μM PVDF membranes. Then membranes were blocked by 5% non-fat milk (in TBST) for at least 1 hour at room temperature. Subsequently, the membranes were incubated with the properly diluted primary antibodies at 4°C overnight. Next day, membranes were incubated with the Horseradish peroxidase-labeled goat anti-mouse or anti-rabbit IgG second antibody for 1h at room temperature. The signals in membranes were visualized with BeyoECL Plus enhanced chemiluminescence reagent (Beyotime Biotechnology, P0018M), and images were collected by gel imaging analysis (Tanon).

### Celltiter-Glo

CellTiter-Glo Luminescence Assay (Promega^®^, G7570) was used to assess the cell viability and determine the IC50 of LASCPC-01 following the treatment of fluoxetine. Cells were plated onto a 96-well plate at 10000 cells/well, then followed by treatment with fluoxetine at different concentrations. After 48 hours of incubation, the cells were lysed by CellTiter-Glo Regent. The luminescent signal produced by ATP in live cells could be measured by a plate reader 10 minutes after lysis.

### Flow cytometry

LASCPC-01 cells were plated in a 10cm dish in HITES culture medium at a concentration of 5×10^6^ cells/dish, and treated with the indicated dose of fluoxetine (dissolved in PBS) at 37°C before collection. Before analysis, cells were washed with PBS and fixed in 70% ethanol in PBS overnight at -20°C. Then the fixed cells were stained with DAPI (10μg/mL) and treated with RNase A (100ng/mL) for 30 minutes at 37°C. BD FACSCanto flow cytometry (BD Biosciences, v3.0) was used to perform cell cycle analysis.

### RNA sequencing

Total RNA was extracted from fluoxetine-treated and PBS-treated LASCPC-01 cells using the TRIzol Reagent (Ambion, 15596026) according to the manufacturer’s protocol. The purity, quantification, and integrity were evaluated using NanoDrop 2000 spectrophotometer (Thermo Scientific) and Agilent 2100 Bioanalyzer (Agilent Technologies). Then the libraries were constructed using VAHTS Universal V6 RNA-seq Library Prep Kit according to the manufacturer’s instructions. The gene expression matrix was uploaded to the Gene Expression Omnibus database GSE224715. Differential expression analysis was performed using the DESeq2. Hierarchical cluster analysis of DEGs was performed using R-software (3.2.0) to demonstrate the expression pattern of genes in different groups and samples. Gene Set Enrichment Analysis (GSEA) was performed using GSEA software to analyze the changed gene sets in the drug-treated group. Significance was set at NES>1.0, *P* value<0.05, and FDR<0.25.

### Animal experiments

All animal experiments in the current study were performed according to the ethics committee of Renji Hospital. Animal experiment protocols were approved by the Renji Hospital Laboratory Animal Use and Care Committee. In order to construct NEPC mice model, genotypes of mice were designed as triple knocked-out (TKO, *PBCre4: Pten^f/f^;Trp53^f/f^;Rb1^f/f^*), as previously reported (8). After 9 weeks, castration was initiated with anesthesia using 1% isoflurane. Surgery was performed on a heating pad until mice completely recovered from anesthesia. All the mice were housed in the same animal room under standard conditions with free access to water (containing antibiotics) and food (standard chow diet). When mice reached the age of 10 weeks, all the experimental mice were randomly divided into two groups for subsequent experiments. Intraperitoneal (i.p.) injections of fluoxetine at 5mg/kg in PBS or a matching vehicle volume per injection, per 2 days, were administered. The body weight was recorded every injection day using an electronic weighing scale. After 4 weeks of injections, the TKO mice were executed by anesthesia, and they were rapidly dissected. The prostate, lung, liver, and lymph nodes were harvested. In addition, for recording the overall survival of TKO mice, the above experiment will be repeated until these mice are moribund. The moribund mice were euthanized, and tumor samples were harvested. Their body weight and viability were monitored, and the date of death was recorded in time.

### Hematoxylin and eosin staining and immunohistochemistry analysis

Tumor tissue specimens were harvested from drug-treated or PBS mice models. The specimens were fixed by 4% paraformaldehyde and embedded in paraffin, then were sectioned as 5μm slices. H&E staining was performed according to routine protocols. For performing Immunohistochemistry analysis, several primary antibodies were used respectively, including SYP, PCNA, and Cleaved-Caspase-3. After DAB reagent detecting expression level, all the slices were sealed with neutral gum. Images of slices were photographed by a light microscope, and the H-score of these images was evaluated by ImageJ software (v2.1.0).

### Glucose and lactate consumption assay

Glucose and lactate concentrations in medium were determined by Glucose Assay Kit-WST (Dojindo^®^, G264) and Lactate Assay Kit-WST (Dojindo^®^, L256), respectively. LASCPC-01 cells were plated in 6-well plates in modified HITES medium. After incubation for 24 hours, cultured media were centrifuged at 1,500 rpm for 5 minutes, and supernatants were collected in 96-well plates in 100μl/well in triplicates. The supernatants were then incubated with corresponding respective reaction buffers at 37°C for 30 minutes, and then the absorbance was measured by a plate reader at 450 nm. The mass of glucose consumption and lactate production was normalized to the average number of cells at the start and end of incubation.

### Lipid deposition assay

Oil Red O staining was performed to assess lipid deposition in neuroendocrine prostate cancer tissues by using Modified Oil Red O Staining Kit (Beyotime^®^, C0158S). Cytospin preparations of LASCPC-01 cells were made by cytocentrifuge, and fixed with 4% paraformaldehyde (PFA) in PBS at 4°C for 1 hour. After staining with Oil Red O staining solution for 15 minutes, cytospin preparations were rinsed with wash solution and counterstained with Hematoxylin Staining Solution (Beyotime^®^, C0107). ImageJ software (v2.1.0)was used to form cell imaging, count cell nuclei, and measure integrated density relative to the cell count.

### Colony formation assay

PC3 cells and PC3-NMYC^ov^ cells were seeded at a low density (1,000 cells/well in 6-well plate) in RMPI-1640 medium, and were left untreated or treated with different concentrations of fluoxetine (500nM, 1μM, 2.5μM, 5μM, and 10μM) for a total of 2 weeks. During the experimental periods, medium (along with fluoxetine) was replaced at regular intervals. After 2 weeks, colonies were fixed with 4% paraformaldehyde and stained with 1% crystal violet for 30 minutes.

### Statistical analysis

All the experiment results were expressed as means ± standard de*vs.*tion (SD) from at least three times independent experiments. Student’s t-test was used to compare two groups (fluoxetine-treated *vs.* control groups). All the data statistical analyses were performed using GraphPad Prism 9.0. P value <0.05 was considered statically significant. *P<0.05, **P<0.01, ***P<0.001, ****P<0,0001.

## Results

### High-throughput drug library screening identified the potential compounds that inhibit the NEPC cell line

**Figure 1:**
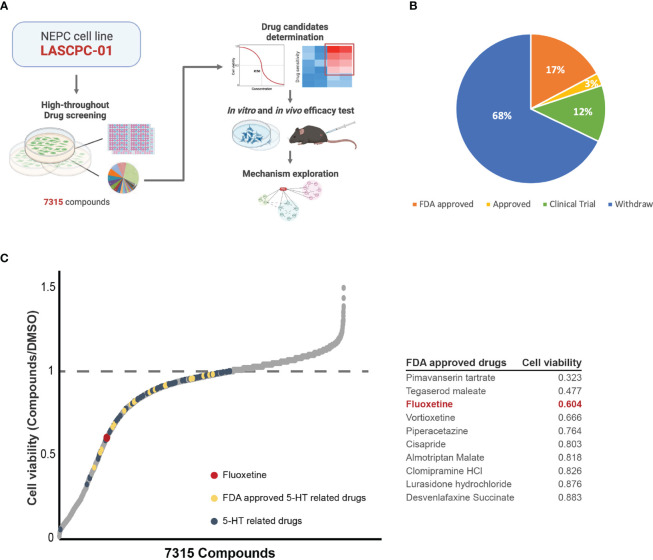
High-throughput drug library screening identified the potential compounds that inhibits the NEPC cell line at the concentration of 2μM. **(A)** Schematic illustration of the whole experiment flow. **(B)** The pie chart of drug library shows the consist of 7315 compounds. **(C)** The effectiveness of each compound in inhibiting NEPC cells. 5-HT related drugs (blue), FDA approved 5-HT related drugs (yellow), and fluoxetine (red) were labelled. And table showing the cell viability of top10 FDA approved 5-HT related drugs.

To identify potential candidates capable of inhibiting the proliferation of NEPC cells, we conducted a high-throughput drug library screening in the NEPC cell line LASCPC-01 (**Figure 1A**). The drug library contains 7315 compounds, among which 17% were FDA-approved drugs (**Figure 1B**). We screened the library at 2μM and monitored it for 72h. Thus, we ranked the drugs according to their inhibitory effect on the LASCPC-01 cell line and found that part of the 5-HT related drugs possessed hopeful tumor suppressive potential and showed a promising effect of tumor suppression (**Figure 1C**). As is shown in **Figure 1C**, we noticed that fluoxetine was the one of top hits in FDA-approved 5-HT related drugs with potential to inhibit the proliferation of LASCPC-01. As one of the most frequently prescribed selective serotonin reuptake inhibitors, fluoxetine selectively targeting to 5-hydroxytryptamine (5-HT) transporter and modulates the concentration of serotonin (9, 10). In addition to being a widely used antidepressant, the effect of fluoxetine on different type of cancer has been investigated. Fluoxetine elicits an anti-cancer response in non-small cell lung cancer, colon cancer, and hepatocellular carcinoma through inducing cell apoptosis and triggering cell cycle arrest (11, 12). In spite of showing potential inhibition on some prostate adenocarcinoma cells, anti-cancer effect and mechanism of fluoxetine in NEPC remain unclear. In the past few decades, several studies have revealed its anti-tumor activity in breast cancer, pancreatic ductal adenocarcinoma, triple-negative breast cancer, and other tumors (13–15). Furthermore, we have also identified several potential drugs that may inhibit the NEPC cell proliferation, including fluoxetine, pimavanserin tartrate, tegaserod maleate, vortioxetine, etc. Given the safety and popularity of fluoxetine in clinical use, we set out to validate its efficacy in several NEPC models and explore the potential mechanism.

### Fluoxetine effectively reduced cell viability and neuroendocrine feature *in vitro*

**Figure 2:**
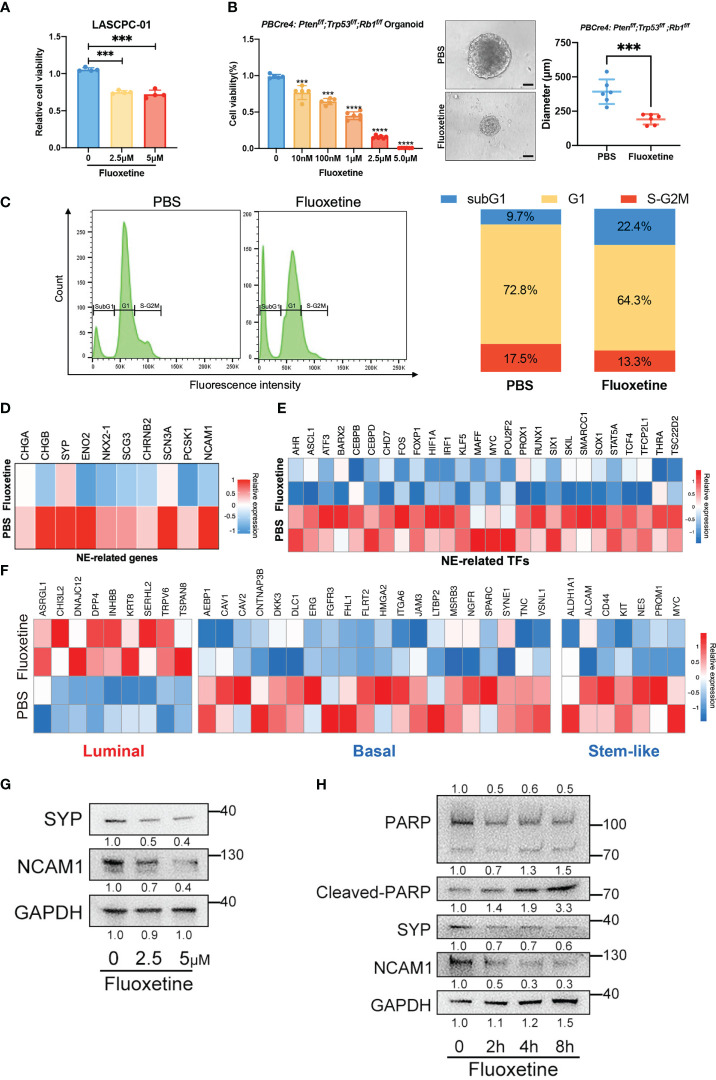
Fluoxetine effectively reduced cell viability and neuroendocrine feature *in vitro.* **(A)** Cell viability of LASCPC-01 after fluoxetine treatment. **(B)** Cell viability and size of organoid derived from *PBCre4: Pten^f/f^;Trp53^f/f^;Rb1^f/f^* mice model tumor in a dose-dependent manner. Representative images were shown. Scale bar, 100µm. **(C)** Cell cycle analysis of LASCPC-01 after 48h treatment with PBS or 5µM fluoxetine (left). Quantification of the sub-G1, G1, and S-G2M fraction were shown (right). **(D)** Heatmap showing the transcriptome analysis of 10 NE-related genes in LASCPC-01 treated with 5µM fluoxetine or PBS. **(E)** Heatmap showing the transcriptome analysis of NE-related transcription factors (TFs) in LASCPC-01 treated with 5µM fluoxetine or PBS. **(F)** Heatmap showing the transcriptome analysis of luminal-related, basal-related, and stem-like in LASCPC-01 treated with 5µM fluoxetine or PBS. **(G)** Western blot showing reduction of NE-related markers after fluoxetine treatment in LASCPC-01 in a dose-dependent manner. **(H)** Western blot showing reduction of NE-related markers and induction of apoptosis markers after fluoxetine treatment in LASCPC-01 in a time-dependent manner. ***<0.001.

To verify the hits from our initial screen, we treated LASCPC-01 cells with different concentrations of fluoxetine. As shown in **Figure 2A**, we confirmed its inhibitory efficiency at both 2.5μM and 5μM. Next, we extended the results in organoids derived from NEPC transgenic mice model (TKO mice, *PBCre4: Pten^f/f^;Trp53^f/f^;Rb1^f/f^*) and found that the fluoxetine reduced both the cell viability and size of organoids in a dose-dependent manner (**Figure 2B**). Further, we performed cell cycle cytometry analysis and found a remarkably increased sub-G1 population, which was inconsistent with the induced cell apoptosis after fluoxetine treatment (**Figure 2C**).

To further investigate the biological effect of fluoxetine on NEPC cells, we performed whole-transcriptome sequencing on LASCPC-01 cells treated with 5µM fluoxetine for 48h. The fluoxetine treatment reduced NE-related gene and transcription factors signature (**Figures 2D, E**). Previous studies reported that the lineage switch of prostate cancer cells from luminal to basal and stem-like cell types contributes to the acquisition of the neuroendocrine phenotype and aggressive variants (16, 17). Consistently, our transcriptome analysis also revealed the upregulation of luminal signature and downregulation of basal and stem-like signature (**Figure 2F**).

Next, we conducted western blot analysis to confirm the reduction of neuroendocrine markers in LASCPC-01 cells treated with fluoxetine for 48h in a dose-dependent manner (**Figure 2G**). In addition, we found reduced neuroendocrine markers and increased apoptosis level (Cleaved-PARP) under the treatment with fluoxetine at 5μM in a time-dependent manner (**Figure 2H**). We treated the prostate adenocarcinoma cells PC3 with fluoxetine and measured the expression levels of NE-related, luminal, basal, and stem-like genes by RT-qPCR. We found that fluoxetine treatment did not significantly alter the expression levels of these genes (**Figure S2A**). In addition, we did colony formation assay after fluoxetine treatment (**Figure S2B**), which showed only weak inhibitory effect on the cell viability of PC3 cells. In the partial neuroendocrine phenotype model (PC3 overexpressed NMYC), with fluoxetine treated, the inhibitory effect of fluoxetine was slightly enhanced (**Figure S2B**). Our findings demonstrated that fluoxetine inhibited cell proliferation and curbed NE phenotype in NEPC cells and organoids.

### Fluoxetine exerts its anti-neuroendocrine effects by inhibiting the AKT pathway

**Figure 3:**
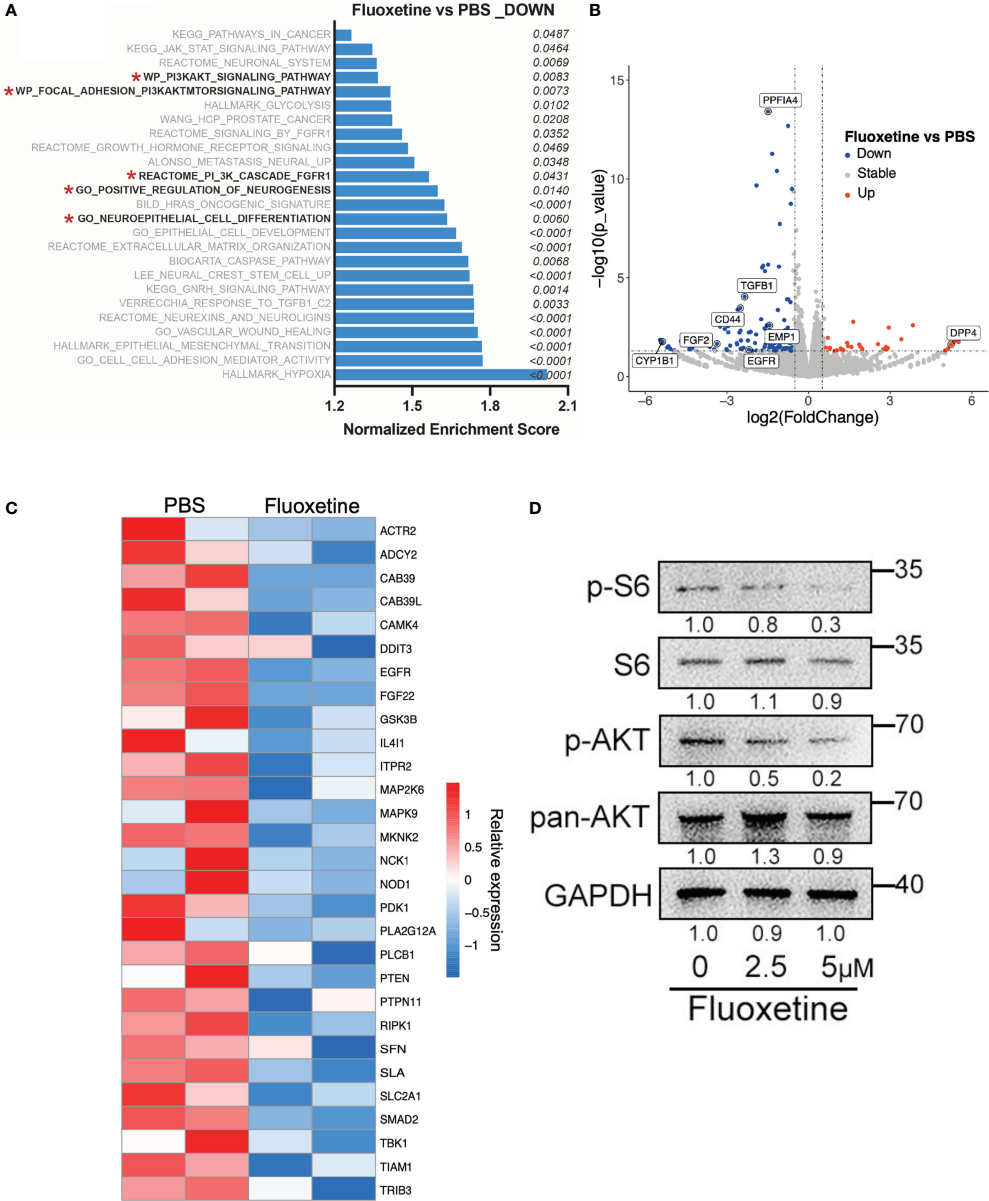
Fluoxetine reduced NE features *via* inhibiting the AKT pathway. **(A)** Gene Set Enrichment Analysis in LASCPC-01 showing pathways downregulated after fluoxetine treatment. **(B)** Volcano plot showing differentially expressed genes in LASCPC-01 after fluoxetine treatment. **(C)** Heatmap showing the transcriptome analysis of AKT downstream genes in LASCPC-01 after fluoxetine treatment. **(D)** Western blot showing the levels of the PI3K-AKT related pathway after fluoxetine treatment in LASCPC-01 in a dose-dependent manner.

We next investigated the potential mechanism responsible for the inhibitory effect of fluoxetine on NE phenotype in prostate cancer. Previous studies have demonstrated that the PI3K-AKT pathway plays an important role in neuroendocrine differentiation of prostate cancer (18). The activation of the AKT pathway plays a crucial role in promoting neuroendocrine differentiation (18–20). Previous study also demonstrated that the combined expression of NMYC and activated Akt1 are sufficient to generated NEPC cell line LASCPC-01 (21). Thus, several therapy strategies targeting the AKT signaling pathway have been developed. MK-2206, a small-molecule AKT pathway inhibitor, showed synergistic anti-tumor effect with chemotherapy including docetaxel, paclitaxel, and platinum in a pre-clinical trial of non-small cell lung cancer (22). Our transcriptome analysis derived from the LASCPC-01 cells suggested that: 1. PI3K-AKT, neurogenesis, and neural differentiation signaling pathway were enriched in fluoxetine-treated cells (**Figure 3A**); 2. the differentially expressed genes analysis identified the downregulation of several PI3K-AKT pathway genes (*EGFR*, *FGF2*), the downregulation of some vital oncogenes (*PPF1A4*, *CYP1B1*, *CD44*, *EMP1*, *TGFB1*), and upregulation of one well-described tumor suppressor gene (*DPP4*) (**Figure 3B**); 3. the reduction of the PI3K-AKT pathway related genes (**Figure 3C**). These results suggested that the PI3K-AKT pathway appears to be affected in LASCPC-01 cells following fluoxetine treatment. We conducted western blot analysis to confirm the inhibitory effect of fluoxetine on the PI3K-AKT pathway. Results from **Figure 3D** showed that phosphorylation levels of AKT and S6 were significantly decreased after the treatment of fluoxetine. In addition, PI3K-AKT pathway has been verified by other studies in other diseases, and this pathway works in almost all the cancers. It is also reported that fluoxetine regulated glucose and lipid metabolism to regulate PI3K pathway (23, 24). We measured Glucose consumption and Lactate production in LASCPC-01 cells treated with or without fluoxetine, and no significant differences were observed across fluoxetine group and control group (**Figures S2C, D**). Next, the lipid metabolism level was explored by Oil red O staining in LASCPC-01 cells. We found that fluoxetine significantly reduced the Oil Red O-positive area (**Figure S2E**). In addition, the lipid metabolism pathway was also reduced as compared with control group (**Figure S2F**). Altogether, our findings demonstrated that fluoxetine reduced lipid metabolism, but not glucose metabolism in NEPC.

### Fluoxetine effectively prolongs the overall survival of NEPC mice and suppresses the neuroendocrine differentiation of tumor tissue

The above cellular experiments on fluoxetine prompted us to expand the research *in vivo*. Specific deletion of *Pten*, *Rb1*, and *Trp53* in prostate epithelial cells developed spontaneous prostate cancer in mice models (8). This TKO mice model showed typical neuroendocrine features: increase of neuroendocrine biomarkers and resistance to androgen deprivation therapy.

**Figure 4:**
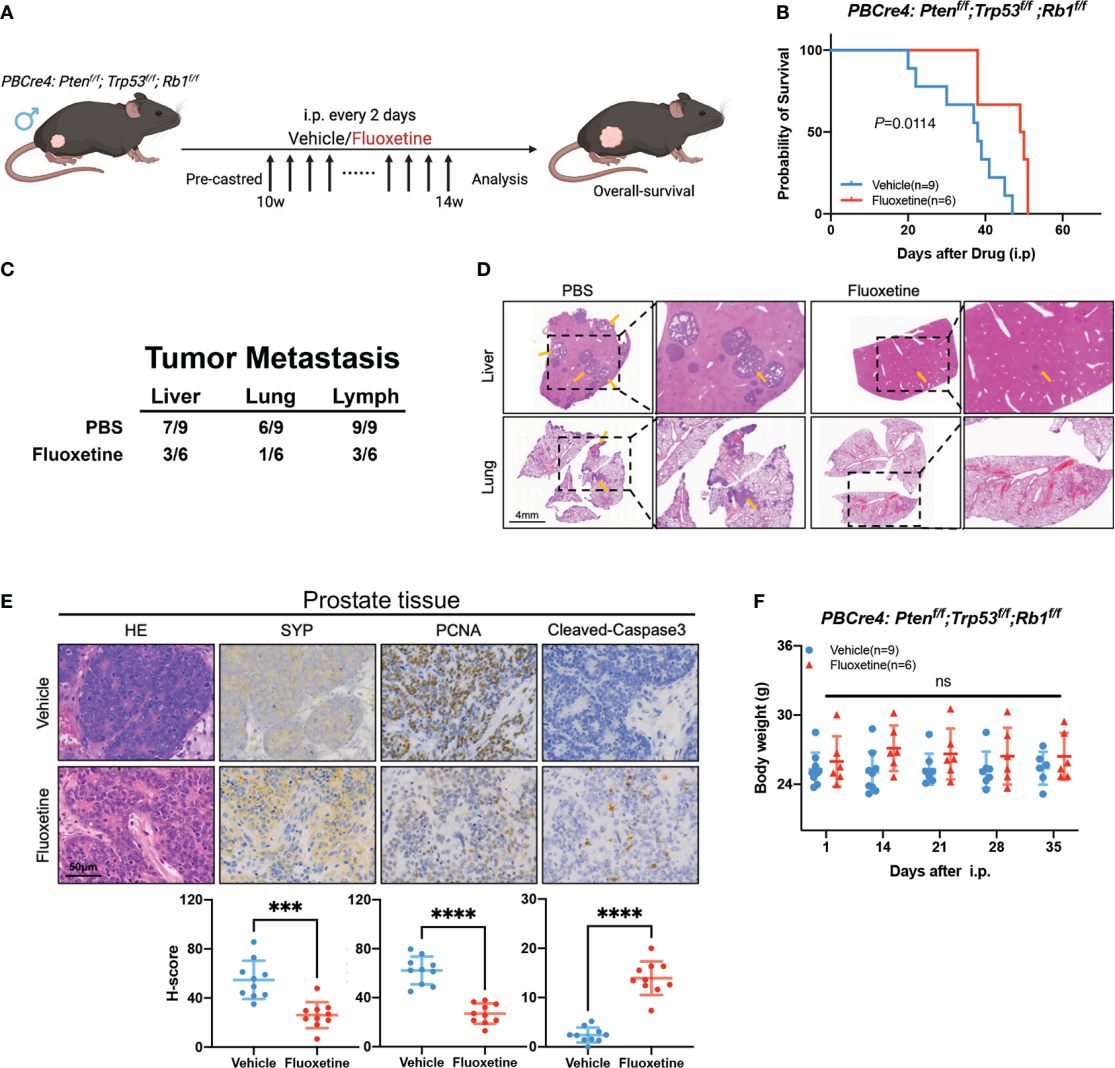
Fluoxetine effectively inhibits tumor growth and prolongs overall-survival in NEPC mice. **(A)** Schematic illustration of the fluoxetine treatment in *PBCre4: Pten^f/f^;Trp53^f/f^;Rb1^f/f^* mice. Mice were injected with fluoxetine or vehicle (i.p.). **(B)** The survival of *PBCre4: Pten^f/f^;Trp53^f/f^;Rb1^f/f^* mice treated with fluoxetine or vehicle. **(C)** Table showing quantifications of tumor metastatic sites in *PBCre4: Pten^f/f^;Trp53^f/f^;Rb1^f/f^* mice treated with fluoxetine or vehicle. **(D)** Representative H&E staining images of mice tumor metastasis (liver and lung). Yellow arrows point to sites of tumor metastasis. Scale bar, 4mm. **(E)** Representative images and quantification of SYP, PCNA, and Cleaved-Caspase3 staining of mice prostate tumor tissue. Scale bar, 50μm. **(F)** Body weight of mice treated with fluoxetine or vehicle. ***<0.001; ****<0.0001; ns, not statistically.

We next examined if fluoxetine application in NEPC genetically engineered mice will prolong survival time and suppress tumor progression. We treated castrated TKO mice with fluoxetine or PBS (**Figure 4A**). Then we monitored the overall survival and body weight of TKO mice. As shown in **Figure 4B**, fluoxetine treatment prolonged the survival of mice (Median survival time: 49.5 *vs.* 38 days). Moreover, fluoxetine reduced the number of metastases in liver, lung, and lymph nodes (**Figures 4C, D**). Subsequent immunohistochemistry analysis revealed that fluoxetine treatment suppressed proliferation and neuroendocrine features of tumors: decreased expression of SYP and PCNA and increased expression of Cleaved-Caspase3 (**Figure 4E**). Body weights of mice were closely monitored, and no significant differences were observed across fluoxetine group and PBS group (**Figure 4F**), suggesting the safety of fluoxetine.

Thus, fluoxetine demonstrated promising application in NEPC genetically engineered mice, such as prolonging survival, suppressing tumor metastasis, decreasing neuroendocrine differentiation, and promoting apoptosis.

## Discussion

NEPC is a lethal subtype of prostate cancer with limited therapeutic approaches, and is featured with poor prognosis and resistance to hormone therapy (2). Currently, cisplatin-based chemotherapy serves as the first-line therapy of NEPC but the clinical outcome remains unsatisfactory (25). We need to explore new effective treatment with good safety. In this study, we performed high-throughput drug screening and identified fluoxetine as a potent drug for NEPC. We demonstrated that the AKT signaling pathway in NEPC cells was disrupted after treatment with fluoxetine. Our results systematically demonstrated the effect of fluoxetine in inhibiting cell viability and promoting apoptosis of LASCPC-01 cells, which provided a theoretical basis for clinical treatment of NEPC.

Currently, drug repurposing is a promising strategy to identify effective drugs for new application. Recent study suggest that the 5-HT pathway plays a key role in mediating neuroendocrine differentiation in advanced prostate cancer (26). Thus, we took 5-HT related drugs into consideration for further investigation. As we know, fluoxetine is a well-known FDA-approved antidepressant prescription and has been wildly used in clinic. It shows good drug safety. According to the drug screening results, it showed excellent inhibition of NEPC cell growth, although it is not the strongest. Although pimavanserin tartrate has shown the strongest anti-cancer effect in high-throughput drug screening (**Figure S1A**), several adverse events such as headache, constipation, and urinary tract infection have occurred in its clinical trials for dementia-related psychosis (27). Considering that NEPC is also a urinary disease, we did not choose pimavanserin tartrate as our first choice. As for tegaserod maleate, compared with the extensive clinical application and numerous clinical studies of fluoxetine, the current clinical research on tegaserod maleate remains limited. We hope to find a drug that is widely used in clinic as a novel treatment strategy for NEPC, so we chose fluoxetine as a candidate drug for follow-up experiments instead of tegaserod maleate.

Previous studied have reported that fluoxetine served as a potential anti-tumor agent in various tumors (13–15). A study in lung cancer revealed that fluoxetine could reduce the proliferation and induce the autophagy of tumor cells by activating the endoplasmic reticulum stress-related protein and the mTOR pathway (28). Besides, fluoxetine was also reported as an effective chemosensitizer, increasing drug accumulation and then slowing down tumor progression (29). In addition, fluoxetine alone or combined with PD-1 blockade demonstrated a long-term control of pancreatic and colorectal tumors (15), which suggested the possibility of combination strategies. As a drug that is already widely used in the clinic, fluoxetine’s safety and reliability are guaranteed. Compared with some other targeted pathway drugs, FDA-approved drugs are able to be promoted and applied in clinical trials more quickly and are more readily available to patients. Like SC66, miransertib, and MK-2206, which target the PI3K-AKT pathway, most of these agents are currently in preclinical studies (22), highlighting the great advantage of FDA-approved drugs. Overall, repurposing FDA-approved drugs for cancer therapy is a more cost-effective and efficient novel strategy for cancer treatment.

The main limitation of our study is that we only use one NEPC cell line LASCPC-01 to confirm the inhibitory effect of fluoxetine. We then applied organoids from NEPC mice tumor to confirm the generalization of our finding. Meanwhile, *in vivo* results from NEPC mice (*PBCre4: Pten^f/f^;Trp53^f/f^;Rb1^f/f^*) supported the antitumor effect of fluoxetine.

## Conclusion

This study performs a high-throughput drug screening in the NEPC cell line LASCPC-01 and identifies fluoxetine as a novel repurposed agent for NEPC therapy. Through further investigation, we revealed that fluoxetine effectively inhibited cell proliferation and neuroendocrine progression. Collectively, we broaden the clinical application of fluoxetine and provide a new insight of drug repurposing strategy for NEPC treatment.

## Data availability statement

The original contributions presented in the study are included in the article/**Supplementary Material**, further inquiries can be directed to the corresponding authors.

## Ethics statement

The animal study was reviewed and approved by the Animal Ethics Committee of Renji Hospital.

## Author contributions

LC, YJ, and AL contributed equally to this work. QW: conceptualization, methodology, writing, revising, supervision, project administration, and funding acquisition. YZ: conceptualization, methodology, revising, supervision, data curation, writing, project administration, and funding acquisition. WX: conceptualization, resources, supervision, project administration. LC, YJ, and AL: conceptualization, methodology, validation, data analysis, data curation, writing, revising and visualization. BL, KS, RS, ZM, WZ: methodology, validation, data analysis, investigation. All authors contributed to the article and approved the submitted version.

## Supplementary material

Supplementary Figure 1 FDA-approved serotonin pathway compounds inhibit the NEPC cell line at different concentrations. **(A)** Cell viability of LASCPC-01 after different serotonin pathway compounds treatment at different concentrations (0.4μM, 1μM, and 2μM)

Supplementary Figure 2 Effect of fluoxetine on neuroendocrine feature and glucose and lipid metabolism. **(A)** RT-qPCR showing the levels of NE-related, Luminal, Basal, and Stem-like genes after fluoxetine treatment (2μM) in PC3 cells. **(B)** Colony formation assays showing the colony formation ability of PC3 and PC3-NMYC^ov^ cells after fluoxetine treatment at different concentrations. **(C)** Glucose consumption was measured in LASCPC-01 cells treated after fluoxetine treatment. **(D)** Lactate production was measured in LASCPC-01 cells treated after fluoxetine treatment. **(E)** Quantitative analysis of Oil red O intensity in LASCPC-01 cells treated after fluoxetine treatment. Scale bar, 50μm. **(F)** RT-qPCR showing the levels of lipid metabolism pathway after fluoxetine treatment (5μM) in LASCPC-01 cells.

Supplementary Table 1 The 5-HT related drugs in high-throughput drug screening.
