# Supplementary figures and images for: High-throughput drug screening identifies fluoxetine as a potential therapeutic agent for neuroendocrine prostate cancer
Source: Front Oncol. 2023 Mar 13;13:1085569. doi: 10.3389/fonc.2023.1085569 (PMC10042075; doi:10.3389/fonc.2023.1085569)

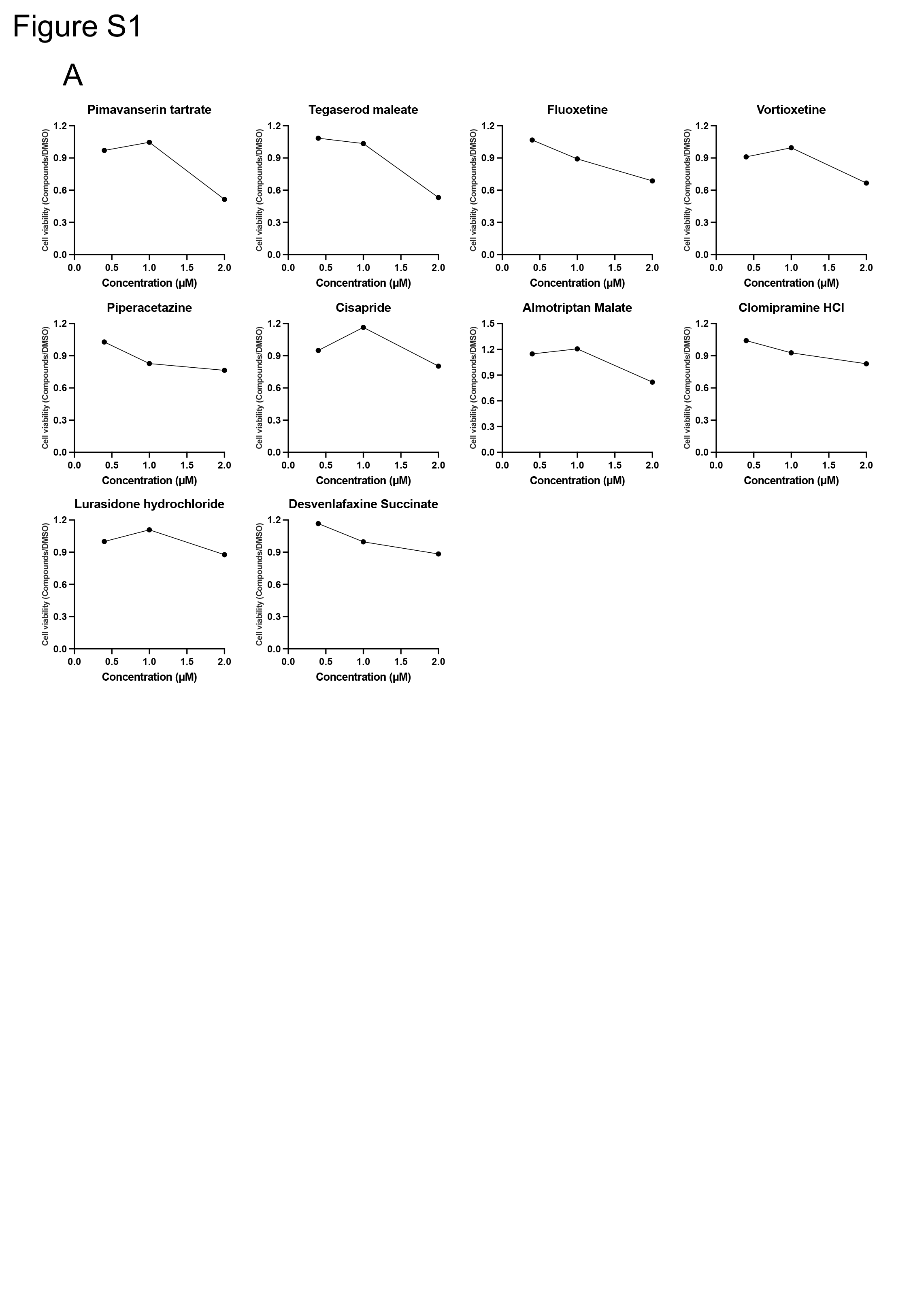

Supplement: Supplementary Figure 1 — FDA-approved serotonin pathway compounds inhibit the NEPC cell line at different concentrations. (A) Cell viability of LASCPC-01 after different serotonin pathway compounds treatment at different concentrations (0.4μM, 1μM, and 2μM) [file Image_1.tif]

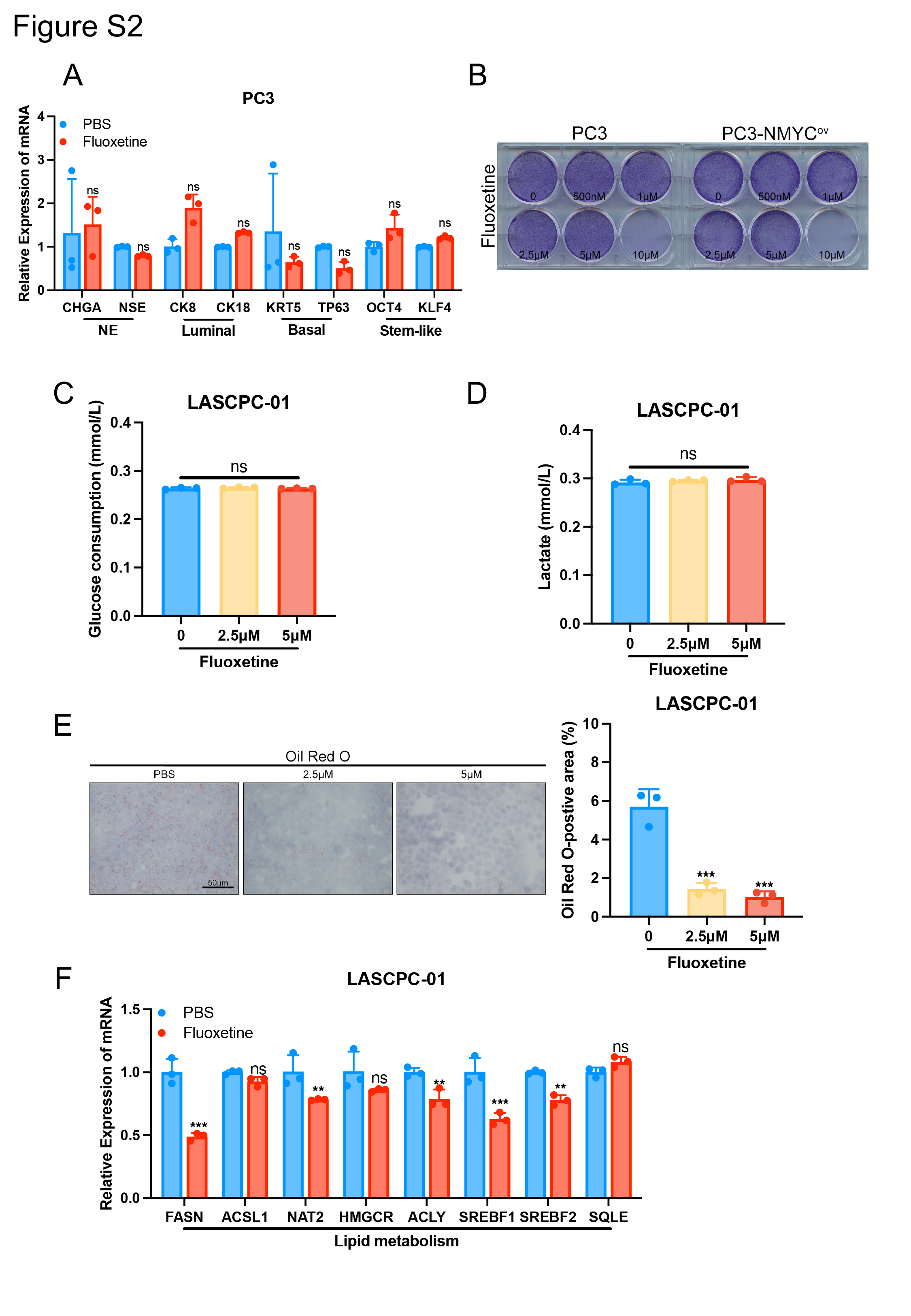

Supplement: Supplementary Figure 2 — Effect of fluoxetine on neuroendocrine feature and glucose and lipid metabolism. (A) RT-qPCR showing the levels of NE-related, Luminal, Basal, and Stem-like genes after fluoxetine treatment (2μM) in PC3 cells. (B) Colony formation assays showing the colony formation ability of PC3 and PC3-NMYCov cells after fluoxetine treatment at different concentrations. (C) Glucose consumption was measured in LASCPC-01 cells treated after fluoxetine treatment. (D) Lactate production was measured in LASCPC-01 cells treated after fluoxetine treatment. (E) Quantitative analysis of Oil red O intensity in LASCPC-01 cells treated after fluoxetine treatment. Scale bar, 50μm. (F) RT-qPCR showing the levels of lipid metabolism pathway after fluoxetine treatment (5μM) in LASCPC-01 cells. [file Image_2.tif]
